# Protein–Ligand Interaction Energies from Quantum-Chemical Fragmentation Methods: Upgrading the MFCC-Scheme with Many-Body Contributions

**DOI:** 10.1021/acs.jpcb.4c05645

**Published:** 2024-11-17

**Authors:** Johannes R. Vornweg, Christoph R. Jacob

**Affiliations:** Institute of Physical and Theoretical Chemistry, Technische Universität Braunschweig, Gaußstr. 17, Braunschweig 38106, Germany

## Abstract

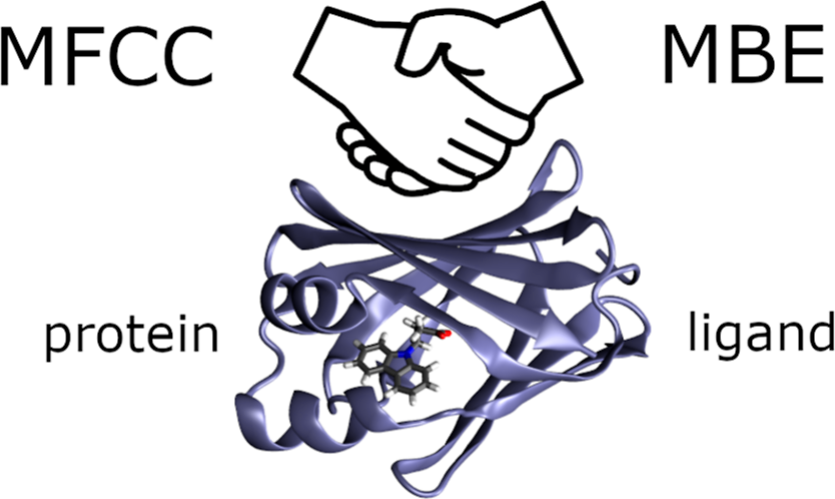

Quantum-chemical fragmentation methods offer an attractive approach for the accurate calculation of protein–ligand interaction energies. While the molecular fractionation with conjugate caps (MFCC) scheme offers a rather straightforward approach for this purpose, its accuracy is often not sufficient. Here, we upgrade the MFCC scheme for the calculation of protein–ligand interactions by including many-body contributions. The resulting fragmentation scheme is an extension of our previously developed MFCC-MBE(2) scheme [*J. Comput. Chem.***2023**, 44, 1634–1644]. For a diverse test set of protein–ligand complexes, we demonstrate that by upgrading the MFCC scheme with many-body contributions, the error in protein–ligand interaction energies can be reduced significantly, and one generally achieves errors below 20 kJ/mol. Our scheme allows for systematically reducing these errors by including higher-order many-body contributions. As it combines the use of single amino acid fragments with high accuracy, our scheme provides an ideal starting point for the parametrization of accurate machine learning potentials for proteins and protein–ligand interactions.

## Introduction

Protein–ligand interactions lie at the heart of drug discovery and molecular biology, playing a crucial role in the design of therapeutically relevant compounds. For accurate estimations of protein–ligand interaction energies, quantum chemistry has emerged as a powerful tool.^1−4^

While methods based on an empirical parametrization of the interaction energy (i.e., classical molecular docking methods) are well established and are routinely used with great success,^5−8^ quantum-chemical calculations can be a valuable complementary tool. First, quantum-chemical methods provide a nonempirical approach which can potentially provide highly accurate interaction energies. Therefore, they can be used to guide the development of empirical energy functions, to validate their accuracy, and to identify their shortcomings (e.g., for partially covalent interactions^9^). Second, they can be applied for nonstandard ligands that contain atoms or structural motifs that are not adequately covered by classical force fields. An important example are metal-based drugs, which are of particular interest as novel anti-infectives (for an example, see ref (10)), but are not routinely amenable to classical docking methods.^11^ Third, quantum-chemical calculations can serve as the basis of accurate machine-learning potentials,^12−14^ which can greatly boost their applicability to large biomolecular systems.

Due to the size of proteins, supermolecular calculations of protein–ligand complexes are usually not feasible with conventional quantum-chemical methods. Quantum-chemical fragmentation methods provide an attractive approach for reducing the required computational effort^15−23^ by dividing the protein into smaller fragments, which can each be treated individually. Several quantum-chemical fragmentation schemes have been adapted for the calculation of protein–ligand interaction energies.^24−34^

While all these approaches offer distinct advantages and are subject to certain limitation, we argue that a fragmentation approach for the calculation of protein–ligand interactions should ideally have the following features: First, it should employ chemically meaningful fragments, such as single amino acids. Not only does this enhance the interpretability of the results and allows for insightful analysis,^35−38^ but it will also provide an ideal staring point for the parametrization of machine-learning potentials.^13,39−42^ Here, the molecular fractionation with conjugate caps (MFCC) scheme,^43−46^ which cuts the protein at the peptide bonds into single amino acid fragments and restores meaningful fragments by capping the severed bonds with acetyl groups (ACE) and *N*-methylamide groups (NME), is particularly attractive (see Section “MFCC for Proteins”). Second, it should provide the possibility for systematic improvement, both in terms of the underlying quantum-chemical methods and in terms of the fragmentation scheme. For the latter, approaches based on a (generalized) many-body expansion (MBE)^34,47−51^ allow for systematically reducing the fragmentation error by including higher-order contributions. Third, it should not only provide access to total energies, but also to other molecular properties, such as the electron density. This will facilitate the inclusion of density-based energy corrections on top of a MBE, as demonstrated in our previous work for molecular clusters.^52−55^ While simpler capping schemes are often sufficient for obtaining accurate energies,^56,57^ the MFCC partitioning has been shown to also provide access to accurate electron densities.^58−60^

We have recently developed a fragmentation scheme that fulfills these three criteria by consistently combining the MFCC scheme using single amino acid fragments with a second-order MBE^61^ (termed MFCC-MBE(2), see Section “MFCC-MBE(2) for Proteins”). Here, we extend this scheme to the calculation of protein–ligand interaction energies, and assess its accuracy for various test cases.

## Computational Methodology

### MFCC for Proteins

**Figure 1:**
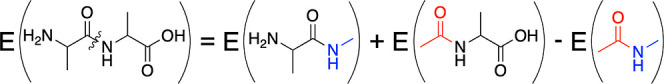
In the MFCC scheme for this alanine dipeptide the peptide bonds are cut and the dangling bonds of the resulting fragments are capped with *N*-methylamide groups (blue) and acetyl groups (red). A new cap molecule *N*-methylacetamide is then formed by the combination of both caps.

In the MFCC scheme,^43−45^ proteins are partitioned into single amino acid fragments by cutting the peptide bonds. To restore dangling valences, the cut bonds are capped with ACE and NME groups (see Figure 1). Disulfide bridges are cut at the S–S bond and capped with methyl sulfide groups.^62^ The capping groups of neighboring fragments are joined to form a cap molecule, which can be subtracted to correct of the effects of the capping groups.

To approximate the total energy of a protein containing *N* amino acids the sum over all energies of the cap molecules, formed by combining the corresponding ACE and NME groups, is subtracted from the sum over all energies of the capped fragments, i.e.
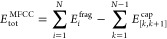
1where *E*_*i*_^frag^ is the total energy of the *i*th capped amino acid fragments, and *E*_[*k*,*k*+1]_^cap^ is the total energy of the cap molecules that results from the cut between amino acids *k* and *k* + 1. For notational simplicity, we do not explicitly show cap molecules resulting from disulfide bridges here and in the following, but these are, of course, handled correctly in our implementation.

### MFCC-MBE(2) for Proteins

The main shortcoming of the MFCC method is that all intramolecular interactions such as hydrogen bonding, which play a crucial role in proteins, are neglected. To alleviate this, several tailored extensions have been proposed, such as using classical electrostatics^63−65^ to correct for missing interactions, the generalized MFCC (GMFCC) method,^66^ which uses additional caps to model the severed hydrogen bonds, or a density-based^67−69^ embedding scheme.

A systematically improvable approach for including interactions between the fragments into quantum-chemical fragmentation methods is provided by the MBE (for a review, see ref (51)). For nonoverlapping molecular fragments, the total energy of a system is first calculated by the sum over all monomer energies followed by the inclusion of all dimer interactions, trimer interactions and so on
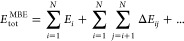
2where *E*_*i*_ is the energy of the *i*-th fragment and Δ*E*_*ij*_ = *E*_*ij*_ – *E*_*i*_ – *E*_*j*_ is the interaction energy between fragments *i* and *j*.

Combining a fragmentation method based on capped or overlapping fragments with the MBE requires carefully ensuring that no double counting occurs, which can be achieved by applying the inclusion–exclusion principle.^70−72^ The most rigorous and general approach is provided by the generalized MBE.^47^ The many overlapping body expansion (MOBE)^48^ is more tractable, but contains some additional approximations.^73^ For a very general reformulation of MBEs for quantum-chemical fragmentation methods, we refer to ref (34).

Recently, we presented a simple and consisted fragmentation scheme that combines the MFCC method with a two-body expansion, termed MFCC-MBE(2).^61^ To ensure that only calculations for the (capped) fragments, caps, and combinations thereof are required, this scheme builds on the MOBE^48^ instead of the more general schemes.^34,47^ The MFCC-MBE(2) method first approximates the total energy by the MFCC scheme, then all fragment–fragment interaction energies are calculated and the sum is added on top of the total energy. To correct for double counting, all occurring fragment–cap and cap–cap interactions are also calculated and enter the equation with a negative sign for the fragment–cap term and with a positive sign for the cap–cap term, i.e.
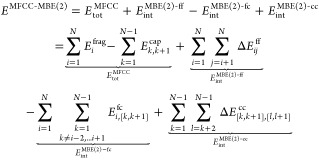
3here, Δ*E*_*ij*_^ff^ is the interaction energy between capped fragments *i* and *j*, Δ*E*_*i*,[*k*,*k*+1]_^fc^ is the interaction energy between capped fragment *i* and cap molecule [*k*, *k* + 1], and Δ*E*_[*k*,*k*+1],[*l*,*l*+1]_^cc^ is the interaction energy between cap molecule [*k*, *k* + 1] and [*l*, *l* + 1].

To make this scheme applicable for single amino acid fragments, three cases for the fragment–fragment interaction energies need to be distinguished
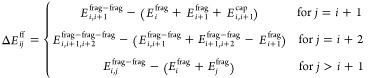
4where the special treatment of the case *j* = *i* + 2 avoids clashes between the capping groups and the neighboring fragment. For further details, we refer to ref (61).

### MFCC and MFCC-MBE(2) for Protein–Ligand Interactions

Protein–ligand interaction energies are obtained as the difference between the energy of the protein–ligand complex *E*^P–L^ and the energies of the separate protein and ligand, *E*^P^ and *E*^L^
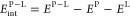
5

The MFCC scheme can be extended to the calculation of protein–ligand interaction energies by replacing each of the terms in eq 1 by the corresponding interaction energy with the ligand. That is, we now calculate the interaction energies of every capped fragment with the ligand and subtract the interaction energies of every cap molecule with the ligand^30,43^
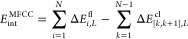
6with
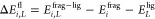
7and
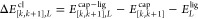
8where *E*_*i*,L_^frag-lig^ and *E*_[*k*,*k*+1],L_^cap-lig^ is the total energy of a supermolecule combining fragment *i* or cap molecule [*k*, *k* + 1] with the ligand.

We note that eq 6 also follows when considering the ligand as an additional (nonbonded) fragment and applying the MFCC-MBE(2) approximation to both *E*^P–L^ and *E*^P^ in eq 5. In this case, all terms that do not involve the ligand cancel, and only those given in eq 6 remain. Thus, eq 6 corresponds to a two-body approximation.

Similarly, the MFCC-MBE(2) scheme can be extended to the calculation of protein–ligand interaction energies by replacing each of the terms in eq 3 by the corresponding interaction energy with the ligand, i.e.
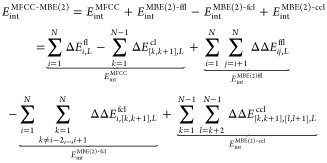
9here, in addition to the MFCC protein–ligand interaction energy *E*_int_^MFCC^ [eq 6], we first have the interaction of two fragments with the ligand, i.e.
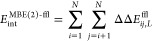
10where we still distinguish the following three cases [cf. eq 4]
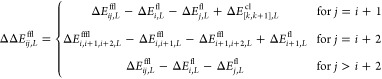
11

In addition, we have the interaction of the fragment–cap combinations with the ligand
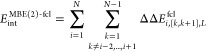
12where
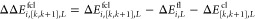
13and the interaction of the cap–cap dimers with the ligand
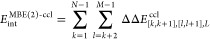
14where
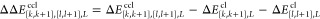
15

Full expressions for each of these terms are given in the Supporting Information.

The above MFCC-MBE(2) approximation to the protein–ligand interaction energy corresponds to a three-body expansion of the total energies of the protein and of the protein–ligand complex. In the Supporting Information, this is further illustrated by showing that the individual terms each have the form of a three-body interaction energy. We note that Antony and Grimme^30^ also give an expression for a three-body approximation to the protein–ligand interaction energy in a MFCC framework [cf. eq 3 in ref (30)]. However, their expression only includes the fragment–fragment terms and misses the cap–fragment and cap–cap contributions. This will lead to a double-counting of intramolecular interaction,^61^ which explains why their three-body correction significantly overcorrects the MFCC fragmentation error for small fragment sizes (cf. Figure 5 in ref (30)).

## Results and Discussion

To assess the accuracy of the MFCC and MFCC-MBE(2) schemes for the calculation of protein–ligand interaction energies, we consider a test set of seven protein–ligand complexes. This test set was assembled by considering examples of protein–ligand complexes previously studied with fragmentation methods (1STP^25,74^) or other computational approaches (2PK4,^75^ 2IFB,^76^ and 1TOW^77−79^). These were complemented with additional test cases (1AAL, 1ABA, 1A6F and 4A4E) that we chose from a search of the protein data bank for protein–ligand complexes featuring protein and ligand sizes that still allow for full quantum-chemical reference calculations.

Our test set consists of a 30–51 disulfide mutant of basic pancreatic trypsin inhibitor in complex with a phosphate ion (PDB-code: 1AAL^80^), the human plasminogen kringle 4 in complex with ε-aminocaproic acid (PDB-code: 2PK4^81^), oxidized bacteriophage T4 glutaredoxin (thioredoxin) in complex with a MES-buffer molecule (PDB-code: 1ABA^82^), streptavidin in complex with biotin (PDB-code: 1STP^83^), ribonuclease P protein in complex with a sulfate ion (PDB-code: 1A6F^84^), the human adipocyte fatty acid binding protein in complex with carbazole butanoic acid (PDB-code: 1TOW^85^) and the rat intestinal fatty-acid-binding protein in complex with palmitate (PDB-code: 2IFB^86^). The size and charge state of each of these test cases are summarized in Table 1. In four cases (1ABA, 1STP, 1TOW, 2IFB), the ligands are deprotonated acids with a single negative charge, while for 2PK4, the ligand is a zwitterion. For 1AAL and 1A6F, the ligands are phosphate and sulfate ions with a negative charge of −3 and −2, respectively.

**Table 1:** Number of Atoms and Charge State of Proteins and Ligands in the Test Set Considered in This Work

	protein		ligand	
PDB	atoms	charge	atoms	charge
1AAL	888	+6	5	–3
2PK4	1203	+5	22	0
1ABA	1397	+2	24	–1
1STP	1744	–2	31	–1
1A6F	1964	+18	5	–2
1TOW	2059	+0	33	–1
2IFB	2113	+0	49	–1

For all proteins, the protonation state is chosen for a realistic pH value (see Section “Computational Details”), resulting in several charged side chains. In these cases, the quantum-chemical calculations for the amino acid fragments do not converge in many cases, unless a continuum solvation model is employed to stabilized the charged amino acid side chains.^30,57^ Here, we employ both a dielectric constant of ε = 4 for the COSMO solvation model (as recommended in ref (57)) and a dielectric constant of ε = 78.39 (corresponding to water). For the ligands carrying multiple negative charges in 1AAL and 1A6F, a dielectric constant of ε = 4 is not sufficient to avoid convergence problems for the fragment–ligand dimers and for fragment–fragment–ligand trimers. In these two cases, MFCC and MFCC-MBE(2) results could only be obtained when using the dielectric constant for water. In all our calculations, we do not apply a distance cutoff in the MFCC part, i.e., the interactions of all fragments and caps with the ligands are included explicitly.

For the MFCC-MBE(2) contribution to the protein–ligand interaction energies, we tested the use of a distance cutoff to reduce the number of trimer calculations that are necessary and thus to reduce the computational effort. To this end, we chose a cutoff λ and include only those fragment–fragment–ligand trimers for which the distances between the ligand and both fragments or for which the distance between the ligand and one fragment as well as the distance between the two fragments are below λ (i.e., at least two of the three intermolecular distances within the trimer are below λ). Here, we define the distance between two molecules as the shortest distance between any of the two fragments’ atoms. The same conditions are used for all fragment–cap–ligand and cap–cap–ligand trimers, respectively.

**Figure 2:**
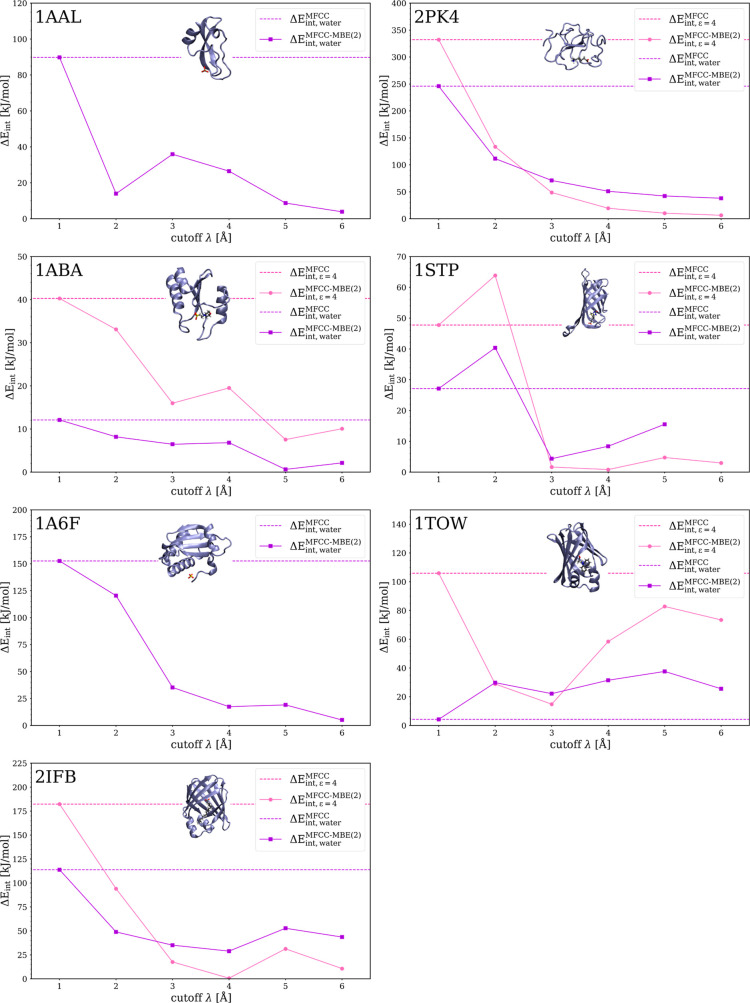
Comparison of the error in the protein–ligand interaction energy (ADF/BLYP/DZP) for the conventional MFCC method and the MFCC-MBE(2) method for a test set of seven protein–ligand complexes. We include results both with a COSMO solvation model using ε = 4 and ε = 78.39 (water).

Figure 2 plots the errors in the protein–ligand interaction energies calculated with MFCC and MFCC-MBE(2) for the seven considered test systems. We calculate this error as
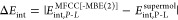
16where *E*_int,P-L_^supermol^ is the protein–ligand interaction energy as obtained from full calculations for the protein–ligand complex, the protein, and the ligand [eq 5] and *E*_int,P-L_^MFCC[-MBE(2)]^ is the approximation to the protein–ligand interaction energy obtained with MFCC [eq 6] or MFCC-MBE(2) [eq 9], respectively. In Figure 2, these errors are plotted as a function of the cutoff λ. The errors in the MFCC protein–ligand interaction energy (for which no cutoff is used) appear as dashed horizontal lines, whereas the errors in the MFCC-MBE(2) protein–ligand interaction energy are shown as solid lines. Note that for λ = 1 Å, all fragment–ligand distances are above this threshold and the results thus coincide with MFCC.

The reference protein–ligand interaction energies calculated in full, supermolecular calculations are listed in Table 2. The largest interaction energies are obtained for the multiply charged sulfate and phosphate ions as ligands (i.e., for 1AAL and 1A6F), where they amount to 3392 and 5046 kJ/mol, respectively. For cases in which the ligands are deprotonated acids with a single negative charge (i.e., for 1ABA, 1STP, 1TOW, and 2IFB), interaction energies between 200 and 600 kJ/mol are obtained, whereas with the zwitterionic ligand in 2PK4, the calculated interaction energy amounts to 683 kJ/mol (for ε = 4) and 794 kJ/mol (for ε = 79.39).

**Table 2:** Errors in the Protein–Ligand Interaction Energy (ADF/BLYP/DZP) for the Conventional MFCC Method and the MFCC-MBE(2) Method for a Test Set of Seven Protein–Ligand Complexes^a^

	1AAL	2PK4	1ABA	1STP	1A6F	1TOW	2IFB
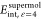		–683.2	–512.4	–323.1		–504.7	–230.4
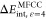		332.3	40.3	47.8		106.0	182.4
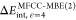		19.5	19.5	0.8		58.4	0.7
*E*_int,water_^supermol^	–3392.1	–794.9	–571.6	–335.0	–5046.4	–567.5	–257.3
Δ*E*_int,water_^MFCC^	89.8	246.0	12.1	27.1	152.6	4.3	113.9
Δ*E*_int,water_^MFCC-MBE(2)^	26.5	51.0	6.8	8.4	17.4	31.5	28.9

a We include results both with a COSMO solvation model using ε = 4 and ε = 78.39 (water). All values in kJ/mol. The MFCC-MBE(2) values are for a cutoff of 4 Å.

The conventional MFCC scheme yields rather crude approximations to the protein–ligand interaction energies in most cases. The largest errors of 332 kJ/mol (for ε = 4) and 246 kJ/mol (for ε = 79.39) are found for 2PK4 (with the zwitterionic ligand). For the cases with multiply charged ligands, the MFCC errors amount to 89 and 153 kJ/mol for 1AAL and 1A6F, respectively. For the test cases in which the ligand carries a single negativ charge, we find MFCC errors between 4.3 and 182.4 kJ/mol. In general, the errors are lower in the calculations using ε = 79.39. The very low error of only 4.3 kJ/mol in the calculations for 1TOW using ε = 79.39 seems to be coincidental, as a rather large error of 106 kJ/mol is obtained for the same system when using ε = 4.

For the calculations of the protein–ligand interactions using our new MFCC-MBE(2) scheme, Figure 2 illustrates the convergence with respect to the chosen cutoff λ. In all cases, it is obvious that the successive inclusion of three-body contributions with increasing cutoff systematically reduces the error of the conventional MFCC scheme. While in most cases, this error reduction is rather monotonic, in some cases (in particular for 1STP, 1TOW and 2IFB) there is some oscillating behavior. Nevertheless, in general the calculated MFCC-MBE(2) interaction energy starts to stabilize at approximately λ = 4 Å. As this value represents a good compromise between convergence of the three-body contributions and the required computational effort, we included the MFCC-MBE(2) values calculated for λ = 4 Å in Table 2.

With MFCC-MBE(2), the errors in the protein–ligand interaction energies are in all cases significantly reduced compared to the conventional MFCC scheme (except for 1TOW with ε = 79.39, where MFCC accidentally shows a very low error). In the calculations with ε = 4, the largest error is obtained for 1TOW with 58 kJ/mol (but for this test case, the error is very dependent on the chosen ε), while in all other cases, MFCC-MBE(2) achieves errors below 20 kJ/mol. In the calculations with ε = 79.39, the largest error is obtained for 2PK4 with 51 kJ/mol, while for all other test cases, the errors are below 32 kJ/mol. When leaving out 1TOW, the error is reduced by at least a factor of 2 in all test cases.

In calculations of protein–ligand interaction energies, one is often interested in relative protein–ligand interaction energies for different conformers in order to identify the correct binding pose of a ligand. Moreover, relative energies present a more challenging test case for quantum-chemical fragmentation schemes, as they probe more subtle effects that might otherwise be masked by large total interaction energies. Therefore, we consider as an additional test case the solution structure of the SMN Tudor domain in complex with symmetrically dimethylated arginine (PDB-code: 4A4E^87^/protein: 971 atoms, charge: −4/ligand: 33 atoms, charge: +1).

**Figure 3:**
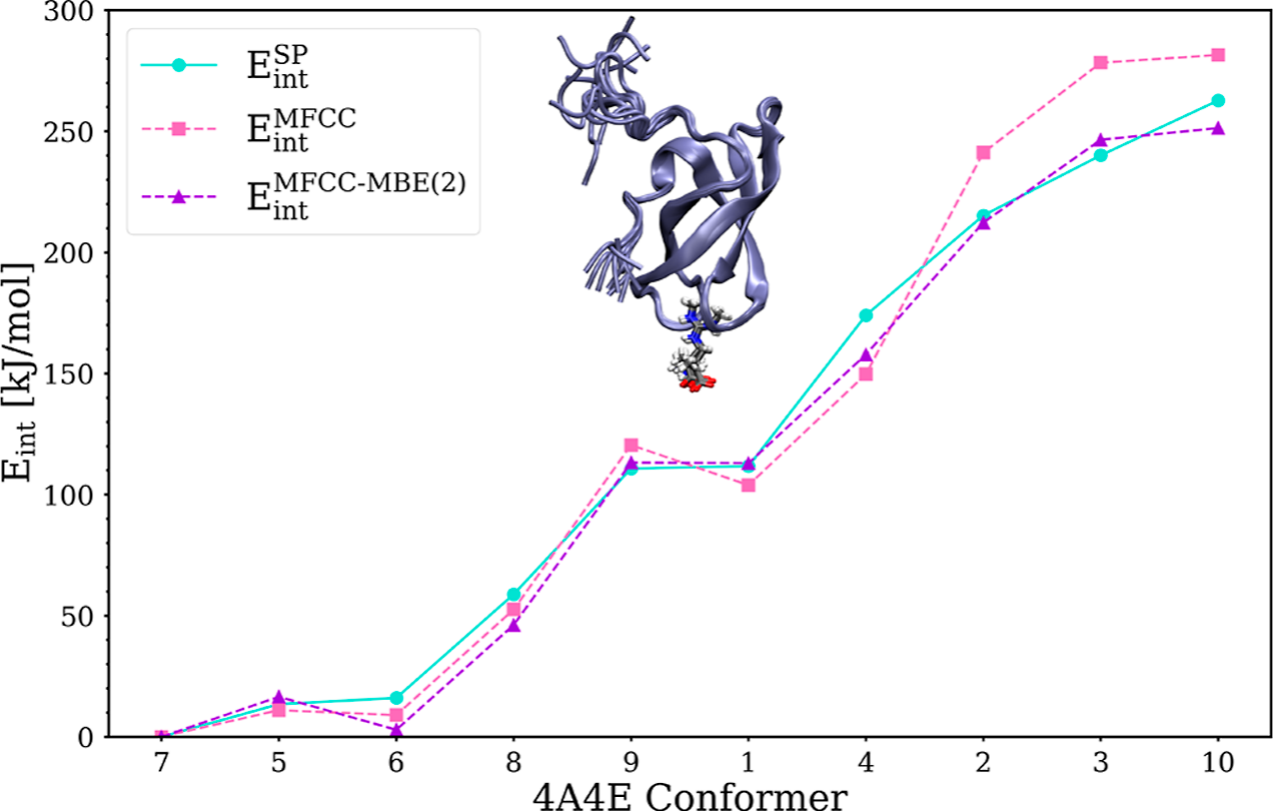
Comparison of the relative interaction energies (ADF/BLYP/DZP) of ten conformers of 4A4E calculated with MFCC (pink) and MFCC-MBE(2) using a distance cutoff of 4 Å (purple) as well as in supermolecular calculations (turquoise). All interaction energies are given relative to conformer 7 and the conformers on the horizontal axis are ordered according to their interaction energy in the supermolecular single-point calculations.

For this test case, the relative protein–ligand interaction energies are shown in Figure 3 relative to the conformer with the largest interaction energy. The conventional MFCC scheme already reproduces the energy pattern rather well, with only small misorder on conformers 6 and 1. The MFCC-MBE(2) scheme has a small misorder at conformer 6 but can recreate the energy order very well. Also the MFCC-MBE(2) scheme does not only get the order right, but it is also closer to the supermolecular results, while the MFCC scheme overshoots especially at the last three conformers 2, 3, and 10.

## Conclusions

We have devised an upgraded MFCC scheme for the calculation of protein–ligand interaction energies. While for the total energy of proteins, the conventional MFCC scheme corresponds to a one-body approximation (i.e., no interactions between fragments are included), we had previously developed the MFCC-MBE(2) scheme for the calculation of the total energy of proteins^61^ that consistently includes two-body contributions.

The conventional MFCC scheme for the calculation of protein–ligand interaction energies^30,43^ corresponds to a two-body approximation if the ligand is considered as an additional fragment. It can be recovered by applying our MFCC-MBE(2) scheme to both the protein and the protein–ligand complex and after removing all terms that cancel in the protein–ligand interaction energy. Here, we have presented an upgraded scheme for the inclusion of three-body contributions in the calculation of protein–ligand interaction energies. While it is equivalent to an MFCC-MBE(3) scheme for the total energy applied to both the protein and the protein–ligand complex, it can be derived straightforwardly by including the ligand in each term of the MFCC-MBE(2) energy.

While in previous work^30^ the error of the MFCC scheme was reduced by increasing the size of the considered fragments, we explicitly set out to develop a fragmentation scheme that operates in terms of single amino acid fragments. This, in turn, necessitates the inclusion of three-body contributions in order to obtain accurate protein–ligand interaction energies. Here, we considered a test set of eight protein–ligand complexes featuring different types of ligands (ranging form multiply charged ions to zwitterionic molecules). We found that while errors in the protein–ligand interaction energies can amount to several hundreds of kJ/mol for conventional MFCC scheme, with the upgraded MFCC-MBE(2) scheme these errors are generally reduced to below 20–30 kJ/mol.

For further reducing these error, we plan to combine the scheme presented here with the density-based many-body expansion,^52^ developed in our group for molecular clusters.^53,54^ Moreover, the efficiency of the calculations can likely be further improved by more refined strategies for screening the two- and three-body contributions. This will open the door to the application of fragmentation schemes in combination with highly accurate wave function-based quantum chemical methods^55^ to proteins and protein–ligand complexes.

Finally, we believe that the MFCC-based many-body fragmentation scheme developed in this work presents an ideal starting point for the development of fragment-based machine-learning potentials for proteins^13,14^ and protein–ligand interactions.

## Computational Details

For all our calculations of protein–ligand interaction energies, the respective PDB structures were used as starting point. For the proteins, the hydrogens were added with PDBFixer 1.9^88,89^ and GROMACS 5.1.4^90,91^ to fit the pH from the original literature (1AAL: pH = 10, 2PK4: pH = 6.0, 1ABA: pH = 6.0, 1STP: pH = 7.8, 1A6F: pH = 7.0, 1TOW: pH = 7.0, 2IFB pH = 7.1). The respective ligands were protonated with Open Babel 3.1.0^92,93^ and manually modified to fit the protonation state in the literature.

An energy minimization was performed with the conjugate gradient algorithm as implemented in GROMACS 5.1.4. The Amber96 force field^94^ was used for the proteins and the GAFF force field^95^ was applied via ACPYPE (AnteChamber PYthon Parser interfacE)^96,97^ to the ligands. In the minimization all heavy atoms were frozen and only the hydrogen atoms were allowed to move. The PDB structures of conformers of 4A4E were already protonated and were not altered. PDB files of all protonated structures used in the calculations presented here are available in the associated data set^98^

The productive runs (calculation of MFCC interaction energies, MFCC-MBE(2) interaction energies, and reference calculations of the interaction energies using the full systems) were performed as using density-functional theory (DFT) calculations with the AMS/ADF program package.^99,100^ We employed the BLYP exchange–correlation functional^101,102^ in combination with a DZP basis set throughout.^103^ Note that for the purpose of assessing the accuracy of the MFCC and MFCC-MBE(2) protein–ligand interaction energies compared to full, supermolecular calculations we did not include a dispersion correction. Dispersion corrections commonly applied in combination with DFT^104^ only depend on the molecular structure and, therefore, the neglect of a dispersion correction does not alter the comparison of the total energies. In all quantum-chemical calculations, we applied the COSMO continuum solvation model^105,106^ as implemented in AMS/ADF.

All calculations were managed via the PyADF scripting framework.^107−109^ The process of constructing the caps has been discussed in our previous publication.^61^ Input scripts for performing the calculations presented here are available in the associated data set.^98^

## Data Availability

Data for this paper, including PDB files of all considered molecular structures, PyADF input scripts for executing the MFCC and MFCC-MBE(2) calculations, and Jupyter notebooks for generating all figures contained in this article, are available at Zenodo at 10.5281/zenodo.13347583 (ref (98)).

## Supporting Information Available

Explicit expressions for the individual terms in the MFCC-MBE(2) protein–ligand interaction energy [eq 9] (PDF)
