# Supplementary material for: Protein–Ligand Interaction Energies from Quantum-Chemical Fragmentation Methods: Upgrading the MFCC-Scheme with Many-Body Contributions
Source: J Phys Chem B. 2024 Nov 17;128(47):11597–606. doi: 10.1021/acs.jpcb.4c05645 (PMC11613497; doi:10.1021/acs.jpcb.4c05645)
Supplement: Supplementary file 1 — jp4c05645_si_001.pdf [file jp4c05645_si_001.pdf]

Protein–Ligand Interaction Energies from Quantum-Chemical Fragmentation Methods: Upgrading the MFCC-Scheme with Many-Body Contributions

Johannes R. Vornweg, Christoph R. Jacob[1]

Technische Universität Braunschweig, Institute of Physical and Theoretical Chemistry,

Gaußstraße 17, 38106 Braunschweig, Germany

Supporting Information

Date: August 20, 2024

[1]E-Mail: c.jacob@tu-braunschweig.de

S1 MFCC-MBE(2) for Protein–Ligand Interactions: Additional Equations

In the MBE of the total energy [cf. Eq. (2)],

$$E_{\text{tot}}^{\text{MBE}} = \sum_{i=1}^{N} E_i + \sum_{i=1}^{N} \sum_{j=i+1}^{N} \Delta E_{ij} + \sum_{i=1}^{N} \sum_{j=i+1}^{N} \sum_{k=j+1}^{N} \Delta E_{ijk} + \ldots, \tag{S1}$$

the trimer interaction energy is given by

$$\begin{aligned} \Delta E_{ijk} &= E_{ijk} - \Delta E_{ij} - \Delta E_{ik} - \Delta E_{jk} - E_i - E_j - E_k \\ &= E_{ijk} - E_{ij} - E_{ik} - E_{jk} + E_i + E_j + E_k \end{aligned} \tag{S2}$$

In the following, we show that the individual terms in the MFCC-MBE(2) protein–ligand interaction energy [cf. Eq. (9)],

$$\begin{aligned} E_{\text{int}}^{\text{MFCC-MBE(2)}} &= E_{\text{int}}^{\text{MFCC}} + \sum_{i=1}^{N} \sum_{j=i+1}^{N} \Delta\Delta E_{ij,L}^{\text{ffl}} - \sum_{i=1}^{N} \sum_{\substack{k=1 \\ k \neq i-2,\ldots,i+1}}^{N-1} \Delta\Delta E_{i,[k,k+1],L}^{\text{fcl}} \\ &\quad + \sum_{k=1}^{N-1} \sum_{l=k+2}^{N-1} \Delta\Delta E_{[k,k+1],[l,l+1],L}^{\text{ccl}} \end{aligned} \tag{S3}$$

result form a three-body expansion of the total energy of the protein–ligand complex, in which the ligand is considered as an additional fragment.

For the fragment–fragment term, we have for $j > i + 2$

$$\begin{aligned} \Delta\Delta E_{ij,L}^{\text{ffl}} &= \Delta E_{ij,L}^{\text{ffl}} - \Delta E_{i,L}^{\text{fl}} - \Delta E_{j,L}^{\text{fl}} \\ &= \left(E_{ij,L}^{\text{frag–frag–lig}} - E_{ij}^{\text{frag–frag}} - E_{L}^{\text{lig}} \right) \\ &\quad - \left(E_{i,L}^{\text{frag–lig}} - E_{i}^{\text{frag}} - E_{L}^{\text{lig}} \right) - \left(E_{j,L}^{\text{frag–lig}} - E_{j}^{\text{frag}} - E_{L}^{\text{lig}} \right) \\ &= E_{ij,L}^{\text{frag–frag–lig}} - E_{ij}^{\text{frag–frag}} - E_{i,L}^{\text{frag–lig}} - E_{j,L}^{\text{frag–lig}} \\ &\quad + E_{i}^{\text{frag}} + E_{j}^{\text{frag}} + E_{L}^{\text{lig}}, \end{aligned} \tag{S4}$$

and for $j = i + 1$,

$$
\begin{aligned}
\Delta\Delta E^{\text{ffl}}_{i,j,L} =& \Delta E^{\text{ffl}}_{ij,L} - \Delta E^{\text{fl}}_{i,L} - \Delta E^{\text{fl}}_{j,L} + \Delta E^{\text{cl}}_{[k,k+1],L} \\
=& \left(E^{\text{frag–frag–lig}}_{i,i+1,L} - E^{\text{frag–frag}}_{i,i+1} - E^{\text{lig}}_{L} \right) - \left(E^{\text{frag–lig}}_{i,L} - E^{\text{frag}}_{i} - E^{\text{lig}}_{L} \right) \\
& - \left(E^{\text{frag–lig}}_{i+1,L} - E^{\text{frag}}_{i+1} - E^{\text{lig}}_{L} \right) + \left(E^{\text{cap–lig}}_{[k,k+1],L} - E^{\text{cap}}_{[k,k+1]} - E^{\text{lig}}_{L} \right) \\
=& \ E^{\text{frag–frag–lig}}_{i,i+1,L} - E^{\text{frag–frag}}_{i,i+1} - E^{\text{frag–lig}}_{i,L} - E^{\text{frag–lig}}_{i+1,L} + E^{\text{frag}}_{i} + E^{\text{frag}}_{i+1} + E^{\text{lig}}_{L} \\
& + \left(E^{\text{cap–lig}}_{[k,k+1],L} - E^{\text{cap}}_{[k,k+1]} - E^{\text{lig}}_{L} \right) \\
=& \ E^{\text{frag–frag–lig}}_{i,i+1,L} - E^{\text{frag–frag}}_{i,i+1} - E^{\text{frag–lig}}_{i,L} - E^{\text{frag–lig}}_{i+1,L} + E^{\text{cap–lig}}_{[k,k+1],L} \\
& + E^{\text{frag}}_{i} + E^{\text{frag}}_{i+1} - E^{\text{cap}}_{[k,k+1]}
\end{aligned} \tag{S5}
$$

for $j = i + 2$, the special treatment involves four-body terms,

$$
\begin{aligned}
\Delta E^{\text{ffl}}_{i,j,L} =& \ \Delta E^{\text{fffl}}_{i,i+1,i+2,L} - \Delta E^{\text{ffl}}_{i,i+1,L} - \Delta E^{\text{ffl}}_{i+1,i+2,L} + \Delta E^{\text{fl}}_{i+1,L} \\
=& \left(E^{\text{frag–frag–frag–lig}}_{i,i+1,i+2,L} - E^{\text{frag–frag–frag}}_{i,i+1,i+2} - E^{\text{lig}}_{L} \right) \\
& - \left(E^{\text{frag–frag–lig}}_{i,i+1,L} - E^{\text{frag–frag}}_{i,i+1} - E^{\text{lig}}_{L} \right) - \left(E^{\text{frag–frag–lig}}_{i+1,i+2,L} - E^{\text{frag–frag}}_{i+1,i+2} - E^{\text{lig}}_{L} \right) \\
& + \left(E^{\text{frag–lig}}_{i+1,L} - E^{\text{frag}}_{i+1} - E^{\text{lig}}_{L} \right) \\
=& \ E^{\text{frag–frag–frag–lig}}_{i,i+1,i+2,L} - E^{\text{frag–frag–frag}}_{i,i+1,i+2} - E^{\text{frag–frag–lig}}_{i,i+1,L} - E^{\text{frag–frag–lig}}_{i+1,i+2,L} \\
& + E^{\text{frag–frag}}_{i,i+1} + E^{\text{frag–frag}}_{i+1,i+2} + E^{\text{frag–lig}}_{i+1,L} - E^{\text{frag}}_{i+1}
\end{aligned} \tag{S6}
$$

Similarly, we find for the fragment–cap terms,

$$
\begin{aligned}
\Delta\Delta E^{\text{fcl}}_{i,[k,k+1],L} =& \ \Delta E^{\text{fcl}}_{i,[k,k+1],L} - \Delta E^{\text{fl}}_{i,L} - \Delta E^{\text{cl}}_{[k,k+1],L} \\
=& \left(E^{\text{frag–cap–lig}}_{i,[k,k+1],L} - E^{\text{frag–cap}}_{i,[k,k+1]} - E^{\text{lig}}_{L} \right) \\
& - \left(E^{\text{frag–lig}}_{i,L} - E^{\text{frag}}_{i} - E^{\text{lig}}_{L} \right) - \left(E^{\text{cap–lig}}_{[k,k+1],L} - E^{\text{cap}}_{[k,k+1]} - E^{\text{lig}}_{L} \right) \\
=& \ E^{\text{frag–cap–lig}}_{i,[k,k+1],L} - E^{\text{frag–cap}}_{i,[k,k+1]} - E^{\text{frag–lig}}_{i,L} - E^{\text{cap–lig}}_{[k,k+1],L} \\
& + E^{\text{frag}}_{i} + E^{\text{cap}}_{[k,k+1]} + E^{\text{lig}}_{L}
\end{aligned} \tag{S7}
$$

and for the cap–cap terms

$$\begin{aligned}\Delta\Delta E^{\mathrm{ccl}}_{[k,k+1],[l,l+1],L} &= \Delta E^{\mathrm{ccl}}_{[k,k+1],[l,l+1],L} - \Delta E^{\mathrm{cl}}_{[k,k+1],L} - \Delta E^{\mathrm{cl}}_{[l,l+1],L} \\ &= \left(E^{\mathrm{cap\text{–}cap\text{–}lig}}_{[k,k+1],[l,l+1],L} - E^{\mathrm{cap\text{–}cap}}_{[k,k+1],[l,l+1]} - E^{\mathrm{lig}}_{L} \right) \\ &\quad - \left(E^{\mathrm{cap\text{–}lig}}_{[k,k+1],L} - E^{\mathrm{cap}}_{[k,k+1]} - E^{\mathrm{lig}}_{L} \right) - \left(E^{\mathrm{cap\text{–}lig}}_{[l,l+1],L} - E^{\mathrm{cap}}_{[l,l+1]} - E^{\mathrm{lig}}_{L} \right) \\ &= E^{\mathrm{cap\text{–}cap\text{–}lig}}_{[k,k+1],[l,l+1],L} - E^{\mathrm{cap\text{–}cap}}_{[k,k+1],[l,l+1]} - E^{\mathrm{cap\text{–}lig}}_{[k,k+1],L} - E^{\mathrm{cap\text{–}lig}}_{[l,l+1],L} \\ &\quad + E^{\mathrm{cap}}_{[k,k+1]} + E^{\mathrm{cap}}_{[l,l+1]} + E^{\mathrm{lig}}_{L} \end{aligned} \tag{S8}$$
